# Model-Informed Precision Dosing of Vancomycin in Hospitalized Children: Implementation and Adoption at an Academic Children’s Hospital

**DOI:** 10.3389/fphar.2020.00551

**Published:** 2020-04-29

**Authors:** Adam Frymoyer, Hayden T. Schwenk, Yvonne Zorn, Laura Bio, Jeffrey D. Moss, Bhavin Chasmawala, Joshua Faulkenberry, Srijib Goswami, Ron J. Keizer, Shabnam Ghaskari

**Affiliations:** ^1^Department of Pediatrics, Stanford University School of Medicine, Palo Alto, CA, United States; ^2^Department of Clinical Pharmacy, Lucile Packard Children’s Hospital Stanford, Palo Alto, CA, United States; ^3^Information Services, Lucile Packard Children’s Hospital Stanford, Palo Alto, CA, United States; ^4^InsightRx, San Francisco, CA, United States

**Keywords:** vancomycin, children, pharmacokinetics, clinical decision support, therapeutic drug monitoring

## Abstract

**Background:**

Model-informed precision dosing (MIPD) can serve as a powerful tool during therapeutic drug monitoring (TDM) to help individualize dosing in populations with large pharmacokinetic variation. Yet, adoption of MIPD in the clinical setting has been limited. Overcoming technologic hurdles that allow access to MIPD at the point-of-care and placing it in the hands of clinical specialists focused on medication dosing may encourage adoption.

**Objective:**

To describe the hospital implementation and usage of a MIPD clinical decision support (CDS) tool for vancomycin in a pediatric population.

**Methods:**

Within an academic children’s hospital, MIPD for vancomycin was implemented *via* a commercial cloud-based CDS tool that utilized Bayesian forecasting. Clinical pharmacists were recognized as local champions to facilitate adoption of the tool and operated as end-users. Integration within the electronic health record (EHR) and automatic transmission of patient data to the tool were identified as important requirements. A web-link icon was developed within the EHR which when clicked sends users and needed patient-level clinical data to the CDS platform. Individualized pharmacokinetic predictions and exposure metrics for vancomycin are then presented in the form of a web-based dashboard. Use of the CDS tool as part of TDM was tracked and users were surveyed on their experience.

**Results:**

After a successful pilot phase in the neonatal intensive care unit, implementation of MIPD was expanded to the pediatric intensive care unit, followed by availability to the entire hospital. During the first 2+ years since implementation, a total of 853 patient-courses (n = 96 neonates, n = 757 children) and 2,148 TDM levels were evaluated using the CDS tool. For the most recent 6 months, the CDS tool was utilized to support 79% (181/230) of patient-courses in which TDM was performed. Of 26 users surveyed, > 96% agreed or strongly agreed that automatic transmission of patient data to the tool was a feature that helped them complete tasks more efficiently; 81% agreed or strongly agreed that they were satisfied with the CDS tool.

**Conclusions:**

Integration of a vancomycin CDS tool within the EHR, along with leveraging the expertise of clinical pharmacists, allowed for successful adoption of MIPD in clinical care.

## Introduction

Vancomycin is a commonly used antibiotic in hospitalized neonates and children, yet it remains challenging to dose in the clinical setting (Hsieh et al., 2014; Brogan et al., 2018). Starting doses commonly recommended for neonates and children frequently fail to achieve desired target exposures (Frymoyer et al., 2013; Ringenberg et al., 2015; Frymoyer et al., 2019; Miloslavsky et al., 2017; Dao et al., 2019). This is due in part to the influence of growth and development on the disposition of drugs which results in large variation in vancomycin pharmacokinetics (PK) between patients (Kearns et al., 2003). In addition, vancomycin has a relatively narrow therapeutic index, and maintaining exposures within a therapeutic window that maximizes treatment benefit, while minimizing potential toxicity, is desirable. Taken together, a standard part of clinical practice during vancomycin therapy is to evaluate exposure and individualize the dose for a patient (i.e. therapeutic drug monitoring [TDM]). To help guide dose individualization during TDM, measurement of a patient’s serum vancomycin concentration is recommended (Rybak et al., 2009; Liu et al., 2011). However, interpretation of drug concentrations is complex and often includes the need for PK calculations along with consideration of patient-specific factors such as age, weight, clinical status, kidney function, dose history, prior drug concentrations, and appropriateness of an assumption of steady-state. Clinical decision support (CDS) tools that can integrate this complex information about a patient within a robust quantitative pharmacokinetic framework that is functional and user-friendly have an opportunity to help advance therapeutic decision-making for vancomycin in children.

Population PK models can serve as a powerful quantitative pharmacokinetic framework to guide therapeutic decision making. By considering patient-specific characteristics that impact drug PK, an individualized dose most likely to achieve the exposure of interest can be calculated. The population PK model-based approach utilizes Bayesian forecasting whereby the model parameters and distribution serve as the Bayesian prior. When new information is gained about the patient (e.g., measurement of drug concentrations in the blood), the system is updated balancing the new information with the Bayesian prior to calculate a posterior probability. A more precise understanding of the pharmacokinetics of a patient is learned, and the dose can be refined to reflect the patient’s pharmacological “signature.” The framework and potential clinical utility of such a model-informed precision dosing (MIPD) approach has been well-described (Sheiner and Beal, 1982; Barrett, 2015; Mould et al., 2016; Darwich et al., 2017; Gonzalez et al., 2017; Neely, 2017). Despite the potential advantages and the recent availability of several MIPD software platforms (Turner et al., 2018), the implementation of MIPD in clinical care has been limited to date (van Lent-Evers et al., 1999; Álvarez-Román et al., 2017>; Abulfathi et al., 2018; Neely et al., 2018; McGann et al., 2019).

In an effort to standardize the therapeutic approach for vancomycin in children at our institution, we recently engaged in a quality improvement (QI) initiative with the aim of improving the process and performance of dose decision-making in patients during clinical care. Central to our evidence-based approach was the implementation of a CDS tool utilizing a MIPD approach. Two central principles guiding our MIPD CDS tool implementation were (1) the need for electronic health record (EHR) integration which provides accessibility within the clinical workflow and minimizes the burden of data-entry to the provider (Kawamoto et al., 2005; Osheroff et al., 2007; Agency for Healthcare Research and Quality, 2009; Miller et al., 2018) and (2) identification of end-users to lead implementation who value and have the clinical need for dose decision-making support (e.g., clinical pharmacists). Herein, we describe our process improvement framework and evaluate the subsequent usage of a MIPD CDS tool for vancomycin in neonates and children at our hospital.

## Methods

### Context

Lucile Packard Children’s Hospital Stanford (LPCH) is a large (350+ bed) freestanding, academic, quaternary-care children’s hospital in northern California, USA with over 13,000 admissions per year. The hospital is divided into separate units including, the Pediatric Intensive Care Unit (PICU), Cardiovascular Intensive Care Unit (CVICU), Neonatal Intensive Care Unit (NICU), Oncology and Stem Cell Transplantation Center, and Acute Care Units. Clinical teams directing care are composed of attending physicians, residents, fellows, nurse practitioners, nurses, and/or clinical pharmacists.

In our hospital, vancomycin is prescribed in neonates and children for suspected or documented infections with methicillin-resistant *Staphylococcus aureus* (MRSA), methicillin-resistant coagulase-negative Staphylococci, and other drug-resistant gram-positive organisms. For invasive MRSA infections - the most common indication for vancomycin - the PK-PD metric associated with outcomes in adults is the vancomycin 24-h area under the curve over the minimum inhibitory concentration (AUC_24_/MIC). Dose individualization to optimize the AUC_24_/MIC are recommended with a target exposure of >400 for invasive MRSA infections (Liu et al., 2011). Due to the practical challenges of calculating this parameter during clinical care, AUC_24_/MIC was not being utilized in therapeutic decision making at our institution and instead trough concentrations were followed. In general, a trough concentration of 10 to 20 mg/L was targeted for suspected/proven MRSA infections, however wide variation between providers in the acceptable target existed. For suspected/proven methicillin-resistant coagulase-negative Staphylococci infections in neonates, a trough concentration of 10 to 20 mg/L was targeted. Within our institution, there was no standardized process for how to perform dose adjustments based on TDM.

Internal audits in 2015 to 2016 as part of ongoing antimicrobial stewardship efforts demonstrated vancomycin was highly utilized in our hospital with an average of 92 days of therapy per 1000 patient hospital days. In those who received vancomycin, > 50% had TDM performed during the course of therapy. Extremely low trough concentrations (< 5 mg/L) were common and occurred in ~30% of patients at the time of first TDM. Taken together, vancomycin management in our hospital is a large clinical workload that is challenging to perform during clinical care. In addition, expanded capabilities are needed to advance our practice in terms of the ability to monitor AUC_24_/MIC.

To develop and implement an updated approach for vancomycin management at our hospital, we formed a multidisciplinary team of physicians (Neonatal and Infectious Disease specialists), clinical pharmacists, and clinical informatic specialists. At the center of the quality improvement initiative was implementation of a MIPD CDS tool to serve as a more convenient, robust, and standardized approach to vancomycin dosing and TDM management at our hospital.

### Model-Informed Precision Dosing Platform

**Figure 1:**
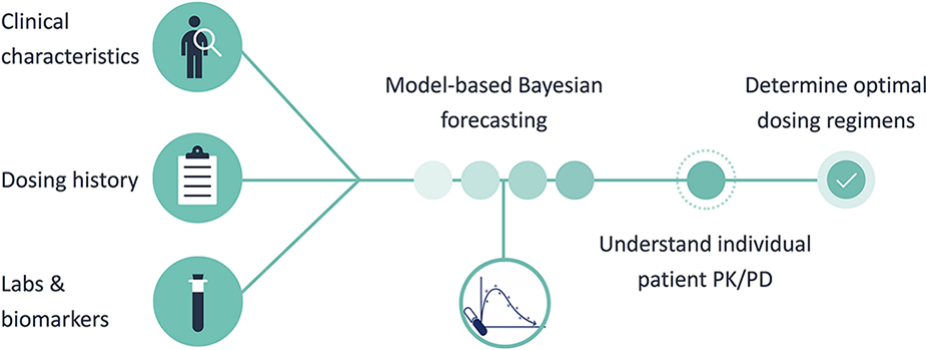
Model-informed precision dosing framework.

MIPD was operationalized using InsightRX, a commercially available, cloud-based precision dosing platform that functions as a CDS tool. The MIPD CDS tool employs *maximum a priori (*MAP) estimation and Bayesian forecasting with a vancomycin population pharmacokinetic model serving as an *a priori* (**Figure 1**). The population pharmacokinetic models available in the MIPD CDS tool are dependent on patient age: Frymoyer et al. (Frymoyer et al., 2014) for patients <52 weeks postmenstrual age versus (herein referred to as neonates) and Le et al. (Le et al., 2013) or Thomson et al. (Thomson et al., 2009) for patients ≥52 weeks postmenstrual age if gestational age known or ≥ 3 months of age if gestational age not known (herein referred to as children). The population PK parameters for each model are presented in **Table 1**. To demonstrate the appropriateness of using the PK models in our patient population, each model was examined in a historical cohort of patients at our institution in terms of the ability to predict vancomycin TDM concentrations collected during clinical care. The bias and precision of each model were assessed by calculating the median prediction error and median absolute prediction error as described by Sheiner and Beal. (Sheiner and Beal, 1981) We previously published our evaluation in neonates (Stockmann et al., 2015; Frymoyer et al., 2019). The pharmacokinetic model by Le et al. was evaluated in n = 86 children (median 4 years [90% range 2 months to 19 years) with n = 190 TDM concentrations. Model predictions had negligible bias (median prediction error 0.2 [95% CI 0.0 to 0.4] mg/L) and adequate precision (median absolute prediction error 1.0 [95% CI 0.7 to 1.3] mg/L) in our population. In addition, 82% of predictions were within 25% of the actual TDM concentration. In the subset of patients 12 years of age or older (n = 23), the model predictions by Thomson et al. were also reasonable (median prediction error 0.5 [95% CI 0.1 to 0.7] mg/L; median absolute prediction error 0.9 [95% CI 0.7 to 1.5] mg/L). Eighty-eight percent of predictions were within 25% of the actual TDM concentration.

**Table 1:** Population pharmacokinetic model parameters of underlying models implemented in the model-informed precision dosing clinical decision support tool.

Model Parameters	Interindividual variability (%)	Residual variability	
		Proportional (%)	Additive (SD)
Frymoyer et al. Model (Frymoyer et al., 2014)			
${\text{CL}}_{\left(\text{L}/\text{H}\right)}=0.345\times {\left(\frac{\text{Wt}}{2.9\text{ kg}}\right)}^{0.75}\times {\left(\frac{1}{{\text{Cr}}_{\text{mg/dL}}}\right)}^{0.267}\times \frac{1}{1+{\left(\frac{{\text{PMA}}_{\text{weeks}}}{34.8\text{ weeks}}\right)}^{-4.53}}$	21.6%	20.5%	1.3 mg/L
${\text{V}}_{\left(\text{L}\right)}=1.75\times \left(\frac{\text{Wt}}{2.9\text{ kg}}\right)$	10.9%		
Le et al. Model (Le et al., 2013)			
${\text{CL}}_{\left(\text{L}/\text{H}\right)}=0.248\times {\text{Wt}}^{0.75}\times {\left(\frac{0.48}{{\text{Cr}}_{\text{mg/dL}}}\right)}^{0.361}\times {\left(\frac{{\text{ln(Age}}_{\text{days}})}{7.8}\right)}^{0.995}$	35%	29%	–
V_(L)_ = 0.636×Wt	18%		
Thomson et al. Model (Thomson et al., 2009)			
CL_(L/H)_ = 2.99×(1+0.0154×(*CrCl*_*ml*/*min*_−66)	27%	15%	1.6 mg/L
V_1 (L/kg)_ = 0.675	15%		
V_2 (L/kg)_ = 0.732	130%		
Q_(L/h)_ = 2.28	149%		

CL, clearance; V, volume of distribution; V_1_, central volume of distribution; V_2_, peripheral volume of distribution; Q, intercompartmental clearance; SD, standard deviation; Wt, weight (kg) PMA, post-menstrual age (weeks); Cr, serum creatinine (mg/dL); CrCl, creatine clearance estimate based on the Cockcroft–Gault equation (ml/min).

**Figure 2:**
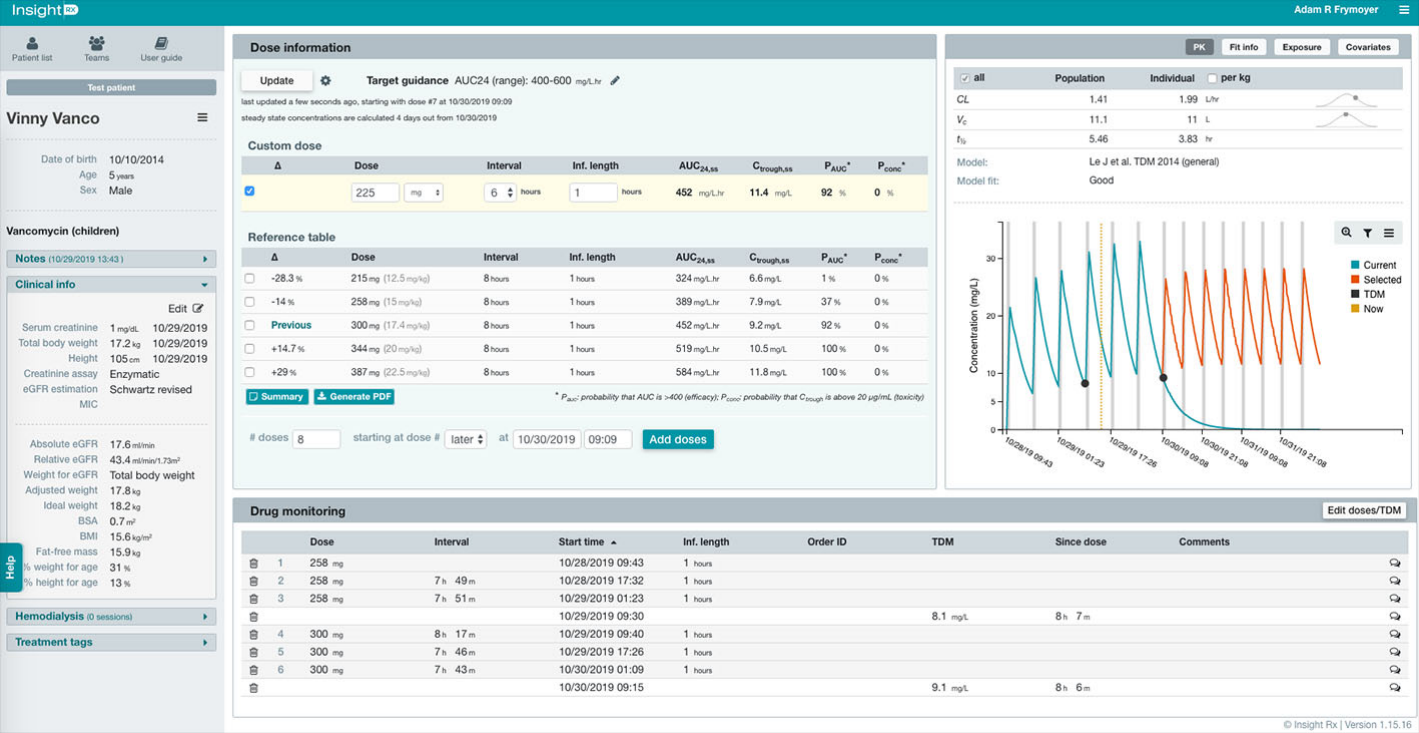
Clinical decision support (CDS) tool user-interface for model-informed precision dosing. Relevant patient characteristics, dose and therapeutic drug monitoring (TDM) history, and model predications are available to user on a single webpage. The user can enter any dosing regimen in the “Custom Dose” table, and the CDS tool will simulate the dosing regimen and estimate the exposure at steady-state for the 24-h area under the curve (AUC_24,ss_) and trough concentration (C_trough,ss_). This process can be repeated to find a dosing regimen most likely to result in the desired exposure.

The user-interface of the MIPD CDS tool, which was optimized based on user testing at Stanford LPCH and other medical institutions, is shown in **Figure 2**. Information needed to support TDM is available to the user in a concise manner within a single web page. This includes the patient’s entire dose and TDM history and characteristics about the patient that impact vancomycin pharmacokinetics such as size (e.g. height, weight, body surface area, etc.), maturation (e.g. chronological age, postmenstrual age, gestational age), and kidney function (serum creatinine and glomerular filtration rate). Users can edit and add any data which may be erroneous or missing from the EHR extraction, and added/edited data are tagged and saved for other users to view. From the patient data, Bayesian estimates of the individual’s pharmacokinetic parameters are calculated and used in simulations to estimate the median steady-state exposure metrics of AUC_24_ and trough at the current dosing regimen (**Figure 2** “Dose Information” -> “Reference Table” -> “Previous”). In addition, the probability of a steady-state AUC_24_ > 400 mg*h/L and a steady-state trough concentration >20 mg/L (representing concern toxic exposure) is calculated taking into account remaining uncertainty in the pharmacokinetic estimates. If a patient is not at the desired exposure, the predicted exposures with doses ± 15% and ± 30% of the current regimen are shown for reference. There is also a unique “Custom Dose” function, where any dosing regimen can be entered by the user and evaluated in terms of the predicted exposure. This process can be repeated to find a dosing regimen for a patient most likely to result in the desired exposure. Other available data in the platform includes individual pharmacokinetic parameter estimates, model fit measures, serum creatinine trend with time, and exposure metrics since the start of treatment.

The cloud-based web app infrastructure of the MIPD platform allows ubiquitous access from any computer within the hospital or even from computers outside the hospital *via* remote login. This accessibility is helpful to provide flexibility and promote adoption. In addition, since cloud-based app no installation or ongoing maintenance of software is required by the local IT department. Finally, the cloud-based infrastructure supports EHR integration.

### Electronic Health Record Integration

The MIPD CDS tool was integrated into our EHR, which supported the functionality of the tool within the clinical workflow and reduced the burden of providers having to manually enter relevant clinical data from the EHR into the tool. To accomplish, we worked with the vendor to design and develop a custom web interface. Our institution had prior experience with EHR integration of web-based CDS tools (Longhurst et al., 2009; Palma and Arain, 2016), which we relied upon as a framework.

**Figure 3:**
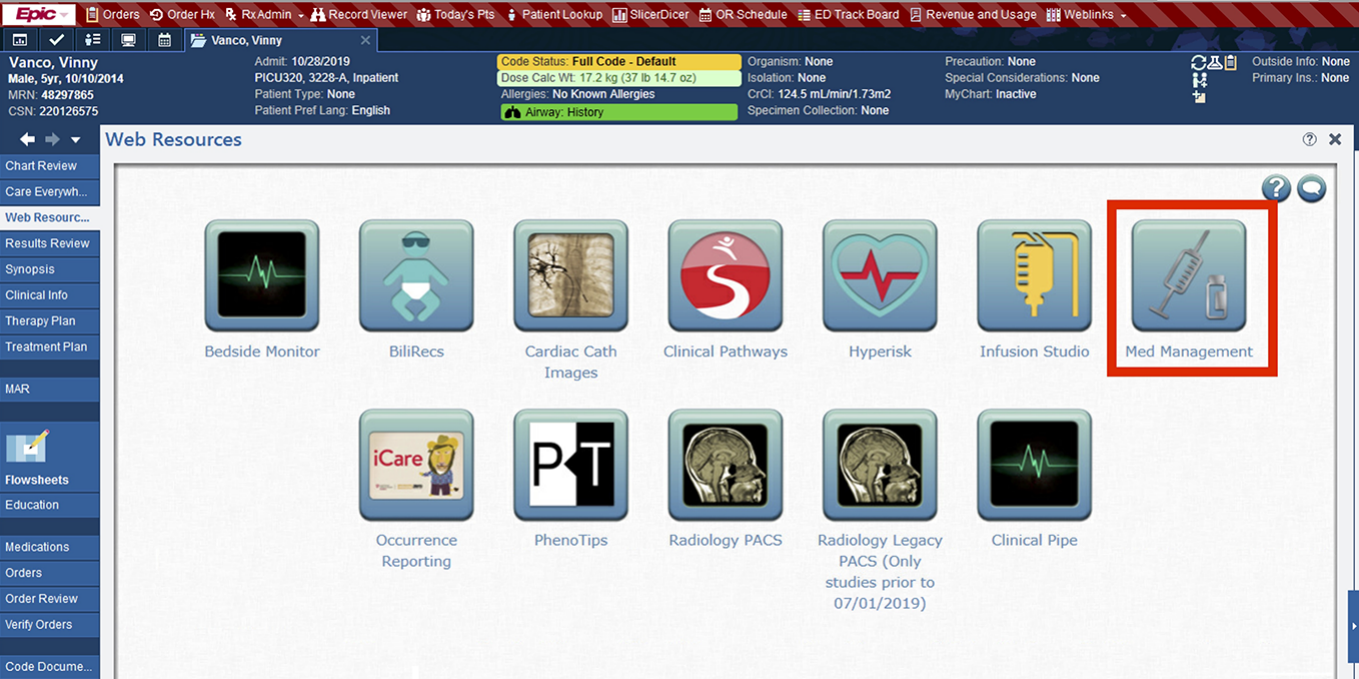
The model-informed precision dosing (MIPD) clinical decision support (CDS) tool is readily accessible within a patient’s electronic health record (EHR) *via* a hyperlink “Med Management” icon. When the icon is clicked, a web browser is opened taking the user to the MIPD CDS tool, and clinical data from the EHR needed for MIPD are securely transmitted to the CDS tool web app.

The integration was accomplished in our EHR (Epic Systems, Verona, WI) *via* a “Med Management” hyperlink icon that displays within the “Web Resources” tab of a patient’s electronic chart (see **Figure 3**). Upon clicking the icon, the MIPD CDS tool (**Figure 2**) is opened in a web browser and a custom application program interface (API) developed by our clinical informatic specialists retrieves all the needed clinical data elements for MIPD from our EHR and compiles them in a JavaScript Object Notation (JSON) file format to be transmitted over a secure (Hypertext Transfer Protocol Secure) connection. Upon receiving the data, the MIPD CDS tool creates a patient specific record, stores the clinical data elements in their Health Insurance Portability and Accountability Act (HIPAA) compliant database, and performs the pharmacokinetic calculations. When new information is available about a patient in the EHR (e.g., additional doses, TDM concentrations, serum creatinine levels), relaunching the MIPD CDS tool from the EHR icon updates the patient’s data and calculations. User authentication is verified using the provider’s EHR user login transmitted *via* the API. Under our EHR integration framework, the provider quickly has access to a patient’s therapeutic relevant information and MIPD output with the total time from launch to guidance taking 5 to 20 seconds, depending on the amount of available historical data for the patient. A standalone version of the MIPD platform is also available and can be accessed *via* any web browser. Patients and data previously transmitted to the platform are available. Any new patients or updates to patient data require manual entry when using the standalone version.

### Implementation of Model Informed Precision Dosing in Clinical Care

At our hospital, clinical pharmacists are integrated members of clinical care teams, routinely follow patients during clinical rounds, and provide medication therapy evaluations and recommendations. In addition, the task of TDM management is often the responsibility of clinical pharmacists including performing pharmacokinetic calculations, which can be time consuming and a burden to clinical workflow. Taken together, clinical pharmacists were uniquely positioned to lead successful adoption of vancomycin MIPD in clinical care.

Prior to implementation in clinical care, all clinical pharmacists received hands-on training on the MIPD CDS tool by representatives of the vendor. They also received didactic training on principles of vancomycin therapy in children including indications, pharmacokinetics, exposure targets, toxicity, and TDM. The didactics included example clinical case scenarios requiring interaction with the MIPD CDS tool. Several clinical pharmacists and members of the multidisciplinary implementation team had previously participated in the vendor’s end-user beta testing of the MIPD CDS tool during development of the design, user interface, and functionality of the tool. Clinical pharmacists on the multidisciplinary implementation team (YZ, LB, JM) were also available to provide frontline support to their peer users.

To facilitate institutional buy-in and commitment, an official policy was developed to formalize the role of clinical pharmacists in managing vancomycin therapy using the MIPD CDS tool. This policy also outlined the process for initiating the clinical pharmacy services during clinical care and set expectations for the clinical care team on clinical pharmacist workflow and responsibilities. To initiate the clinical pharmacist-led MIPD approach, a “Vancomycin per Pharmacy” order was placed in the EHR by the treating physician. The decision to utilize this approach was left to the discretion of the treating physician.

Concurrent with MIPD implementation, an institutional vancomycin dosing and monitoring guideline for neonates and children was developed to help serve as a resource for clinical pharmacists. The guideline provides recommendations on vancomycin starting dose which for children was 15 mg/kg every 6 h or 8 h with lower dosing if reduced kidney function was present (i.e. estimated glomerular filtration rate < 75 ml/min/1.73m^2^) (Frymoyer et al., 2009; Frymoyer et al., 2010; Liu et al., 2011). At our hospital, estimated glomerular filtration rate is calculated using the modified Schwartz equation (Schwartz et al., 2009). The starting dose for neonates was based on a MIPD approach using weight, PMA, and serum creatinine (Frymoyer et al., 2019). TDM was recommended for all patients anticipated to be on vancomycin for >48 h and consisted of a trough concentration before the fourth dose or sooner for those with impaired or unstable renal function. The vancomycin exposure target was flexible and was individualized for each patient in discussion with the clinical care team. If AUC_24_/MIC was used, a steady-state target of >400 was suggested, while if trough concentration was used, a target of 10 to 15 mg/L (or 15–20 mg/L for meningitis, osteomyelitis, or endocarditis) was suggested. The flexibility in exposure target was necessary to gain buy-in across providers at our institution not ready to commit to a strictly AUC_24_/MIC based target. An MIC of 1 mg/L was assumed for AUC_24_/MIC calculations as this value equals the MIC_90_ for MRSA at our institution based on broth microdilution. The MIC was updated if MRSA was isolated on culture in a patient. In addition, for non-MRSA infections, such as coagulase-negative *Staphylococcus*, the most common indication for vancomycin in our NICU population, the importance of AUC_24_/MIC based on pharmacokinetic/pharmacodynamic considerations has not been established.

Prior to clinical use, quality control of the EHR integration was performed in approximately 25 patients over a three month beta testing phase to confirm the accuracy and completeness of patient data transmission from the EHR to the MIPD CDS tool. The MIPD CDS tool was not used for patient care during beta testing. After beta testing of the CDS tool, the clinical pharmacist led MIPD approach was implemented in clinical care in a stepwise fashion starting with the NICU as a pilot in May 2017. The NICU was chosen as it is a highly structured and contained unit in which to roll-out a new CDS tool. Further, several team members work clinically in the NICU including a physician (AF) and clinical pharmacist (YZ), which allowed for hands-on support and assistance in the pilot phase. After a successful pilot phase, the clinical pharmacist-led MIPD approach went live in the PICU in September 2018. The clinical pharmacist lead for the PICU was also an advocate for the MIPD approach and became an expert user through training and hands on use of the MIPD CDS tool. Over the next 14 months, experience and comfort using the MIPD CDS tool was gained by the clinical pharmacists. Minor refinements to the usability of the MIPD CDS tool and workflow processes of the clinical pharmacist were made. Overall, user feedback was positive (see clinical pharmacist survey below), and in November 2018, the MIPD approach was expanded to the entire hospital.

### Clinical Use and User Satisfaction of Model-Informed Precision Dosing

The MIPD platform serves as a central database containing information on patients at our hospital for whom the MIPD CDS tool was used. This offers the ability to track metrics around usage and patient characteristics during our quality improvement initiative. In addition, summary level exposure metrics such as predicted steady-state trough concentration and AUC_24_ achievement were available within the MIPD analytics platform using patient clinical characteristics, dose history, and TDM levels. Herein, we summarize our MIPD usage over the period of May 2017 (start of implementation) to June 2019. For patients who received multiple courses of vancomycin (a separate course defined as >14 days between vancomycin doses), each course was counted as a separate patient-course. For comparison, the number of vancomycin patient-courses at our hospital was examined over the same time period through a query of our EHR, which captures patient-courses regardless of MIPD usage. We then calculated the proportion of patient-courses at our hospital each month for which MIPD was utilized. Demographic and PK data did not follow normal distributions when examined using the Shapiro-Wilk test (p < 0.05). Therefore, descriptive statistics were calculated as median (interquartile range) or counts (%).

To assess satisfaction, perceived usability, and overall clinical experience with the MIPD CDS tool, clinical pharmacists were invited *via* email to participate in a survey 15 months after initial clinical implementation in the NICU and PICU (August 2018). The survey gave users a more formal mechanism to share feedback on the MIPD CDS tool that could be used to help inform and update our quality improvement processes around implementation of the MIPD CDS tool. The survey was administered online using the web application REDCap (Research Electronic Data Capture) hosted at Stanford University (Harris et al., 2009). The survey was adapted in part from a validated questionnaire (Post-Study System Usability Questionnaire) measuring satisfaction with the usability of a system with responses in the format of a Likert scale (Lewis, 2002). In addition, questions related to the number of patients treated and importance of specific features were also included.

This project was reviewed by Stanford University’s institutional review board and determined to be local quality improvement work that did not meet the definition of human subjects research (U.S. Department of Health and Human Services (HHS) regulations at 45 CFR part 46). The need for written informed consent was, therefore, waived.

## Results

### Clinical Use

During the first 2+ years of clinical implementation (May 2017 to June 2019), a total of 853 patient-courses (n = 96 neonates, n = 757 children) comprising 7,800 doses and 2,148 vancomycin TDM levels were evaluated by clinical pharmacists within the MIPD CDS tool. Characteristics of patient-courses in which the MIPD CDS dosing tool was utilized are shown in **Table 2**. TDM was performed in 88% (750/853) of patient-courses for which the CDS tool was utilized.

**Table 2:** Characteristics of patient courses in which the model informed precision dosing CDS tool was used during vancomycin treatment (May 2017 to June 2019).

	Neonatal Platform^a^ (n = 96)	Child Platform^b^ (n = 757)
Age, years	44 (21–76) days	6.0 (1.7–13.4) years
Gestational age, weeks	39.3 (35.4–40.0)	–
Weight, kg	3.1 (2.2–3.9)	19.1 (9.7–42.6)
Height, cm	49.0 (42.4–54.0)	105 (73–148)
Female, *n* (%)	34 (35%)	338 (44%)
Serum creatinine, mg/dL	0.3 (0.2–0.6)	0.4 (0.2–0.6)
Estimated Glomerular Filtration Rate^c^, ml/min/1.73 m^2^	–	137 (101–182)
Duration of treatment course, days	7 (2–12)	6 (2–13)
TDM^d^ per treatment course, n (%) 0 1 2 3 ≥4	13 (14%) 30 (31%) 23 (24%) 14 (15%) 16 (17%)	62 (8%) 293 (39%) 147 (19%) 104 (14%) 151 (20%)

All data are median (interquartile range) or number of patients (%).

a Patients <52 weeks postmenstrual age.

b Patients ≥52 weeks postmenstrual age if gestational age known or ≥ 3 months of age if gestational age not known.

c Calculated using modified Schwartz equation (Schwartz et al., 2009).

d TDM, therapeutic drug monitoring concentrations.

**Figure 4:**
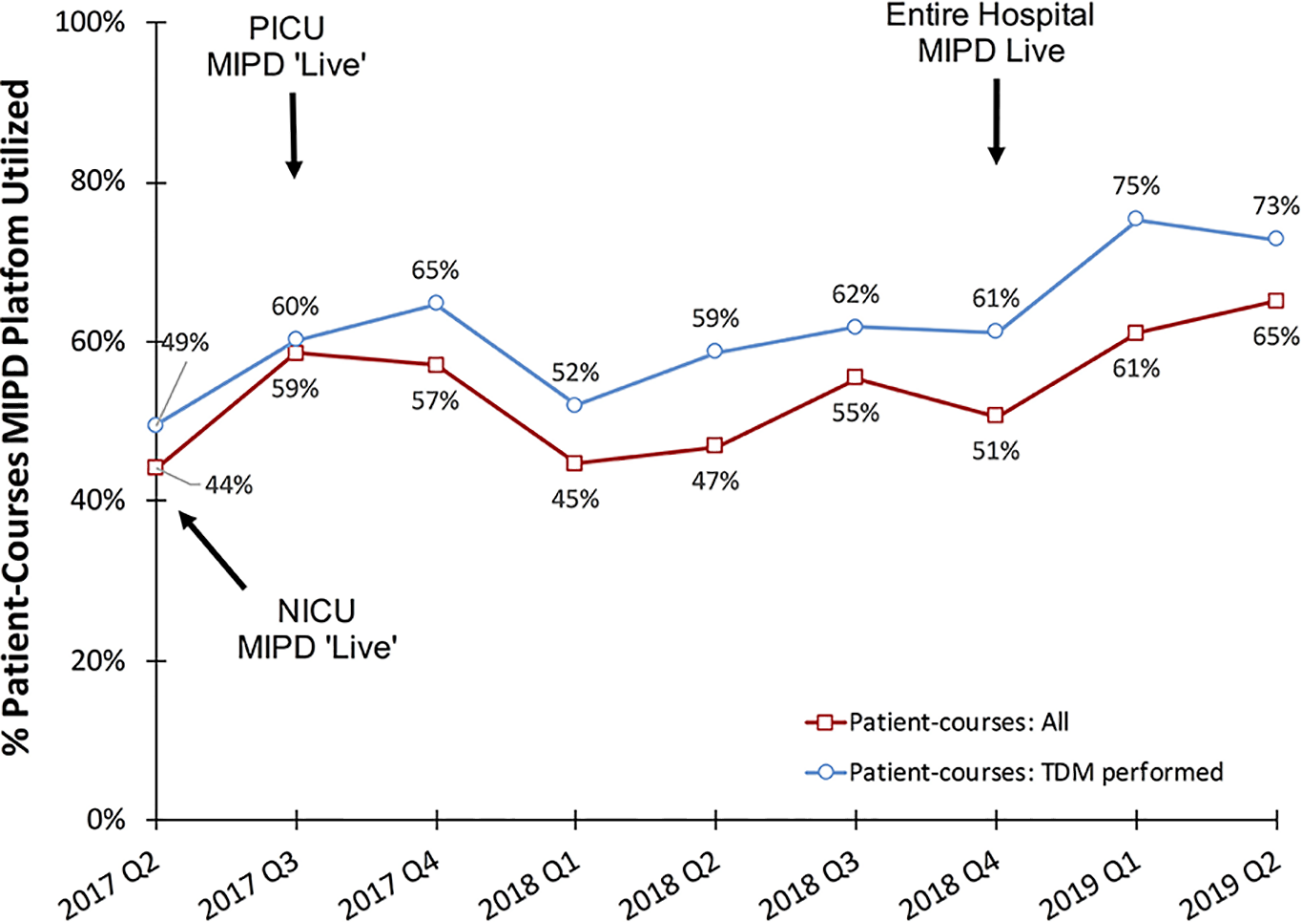
Percentage of vancomycin patient-courses utilizing the MIPD CDS tool by quarter. “Patient-courses: All” are courses of vancomycin where at least one dose was given. “Patient-courses: TDM performed” are courses of vancomycin where at least one therapeutic drug monitoring concentration was measured.

Among all vancomycin patient-courses at our hospital the percentage in which the MIPD CDS tool was utilized is shown by month in **Figure 4**. During the entire clinical implementation time-period, the MIPD CDS tool was utilized to support 54% (853/1587) vancomycin patient-courses at our hospital and 62% (750/1217) of patient-courses in which TDM was performed. In the most recent 6 months since hospital-wide availability of the clinical pharmacist led MIPD approach (January 2019 to June 2019), the MIPD CDS tool was utilized to support 63% (202/320) of vancomycin patient-courses and 74% (170/230) of patient-courses in which TDM was performed.

Vancomycin exposure in patients who had TDM performed are shown in **Table 3**. Extreme trough concentrations as measured by a predicted steady-state trough <5 mg/L or >20 mg/L were low with 87.8% (64/82) of neonates and 79.0% (528/668) of children having achieved a predicted steady-state trough concentration between 5 and 20 mg/L by the second TDM. AUC_24_/MIC >400 at steady-state was achieved in 63.4% (52/82) of neonates and 46.7% (312/668) of children at the time of first TDM, and this improved to 78.0% (64/82) of neonates and 64.1% (428/668) of children by the second TDM. If a target predicted trough concentration of 10 to 20 mg/L was used, 53.7% (44/82) of neonates and 44.3% (296/668) of children achieved this target by the second TDM.

**Table 3:** Vancomycin exposure in patients who had therapeutic drug monitoring and the MIPD CDS tool was used.

	Neonate Platform			Child Platform		
	TDM #1	TDM #2	TDM #3	TDM #1	TDM #2	TDM #3
AUC_24,ss_ (mgxh/L)	433 (362–525)	426 (367–523)	428 (353–32)	406 (308–530)	421 (318–548)	451 (338–579)
Cumulative Achievement of AUC_24,ss_/MIC >400 (%)^a^	63.4%	78.0%	78.0%	46.7%	64.1%	70.1%
Trough,_ss_ (mg/L)	9.2 (6.7-13.0)	9.3 (7.0-13.7)	8.5 (7.0-12.0)	8.8 (5.5-13.2)	9.3 (5.6-13.8)	10.4 (6.4-15.3)
Cumulative Achievement of Trough_,ss_ 5 to 20 mg/L (%)^b^	78.0%	87.8%	90.2%	60.5%	79.0%	82.6%
Cumulative Achievement of Trough_,ss_ 10 to 20 mg/L (%)^c^	41.5%	53.7%	56.1%	28.1%	44.3%	47.3%

AUC_24,ss_/MIC, 24-h AUC over the MIC at steady-state assuming MIC = 1 mg/L; Trough_ss_, predicted trough concentration at steady-state; TDM #1 represents predicted exposures before any dose adjustments; TDM #2 represents predicted exposures after the first dose adjustment; TDM #3 represents predicted exposures after the second dose adjustment.

a Cumulative percentage of patients who achieved AUC_24,ss_/MIC >400 over the first three TDM evaluations.

b Cumulative percentage of patients who achieved a predicted trough of 5 to 20 mg/L over the first three TDM evaluations.

c Cumulative percentage of patients who achieved a predicted trough of 10 to 20 mg/L over the first three TDM evaluations.

### Clinical Pharmacist Satisfaction and Experience

**Figure 5:**
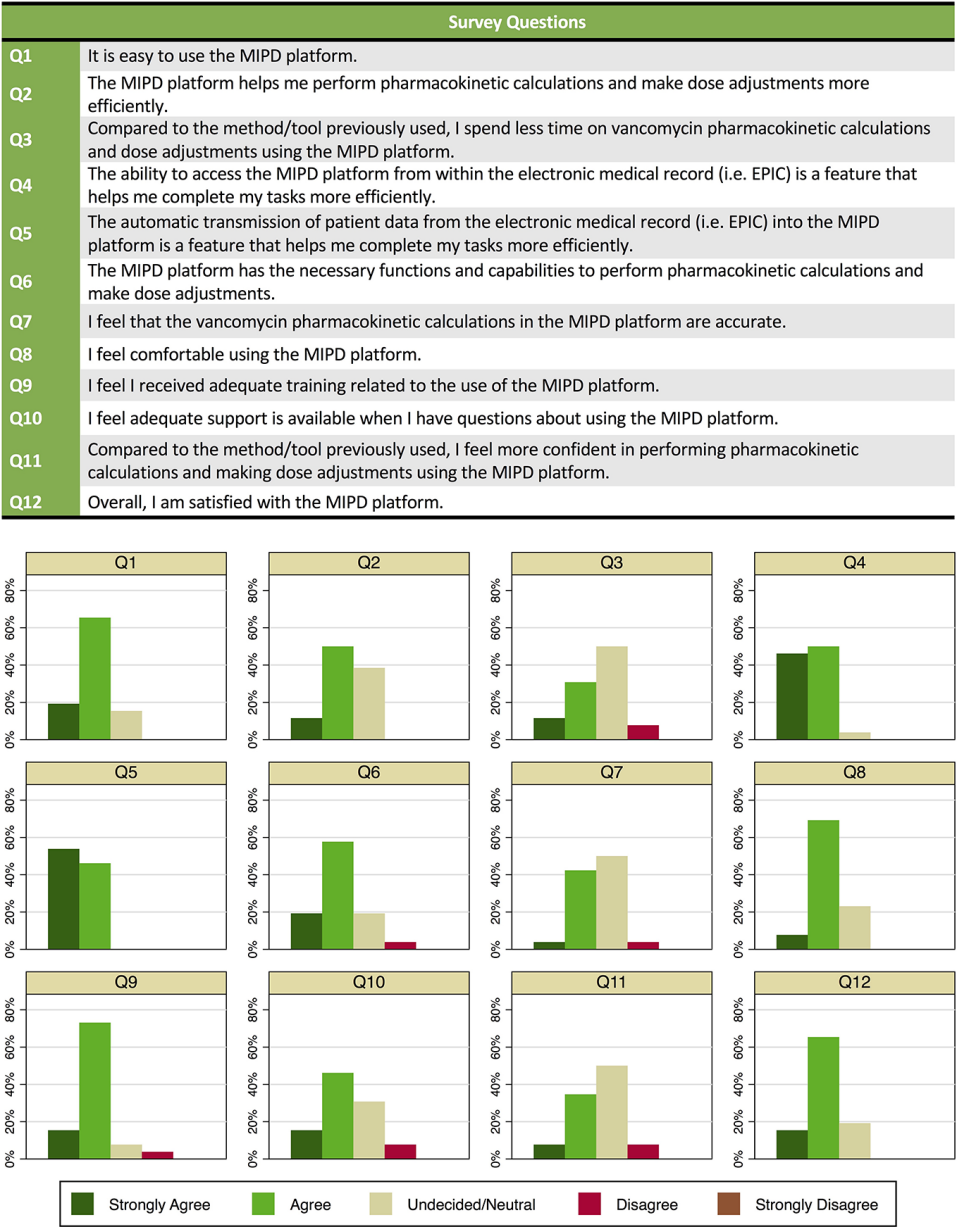
Survey questions and responses of clinical pharmacists (n = 26) on use of and experience with the model-informed precision dosing clinical decision support (CDS) tool in clinical care.

Of 63 clinical pharmacists at our hospital, 46 had used the MIPD CDS tool in clinical care over the first 15 months of implementation, and of those 26 participated in a survey about their use and experience. Of the 26 survey participants, 8 (37%) reported using the tool in >20 patients, 9 (35%) had used in 6 to 20 patients, and 9 (35%) had used in ≤ 5 patients. Of the 26 clinical pharmacists who completed the survey, 21 (81%) noted dose individualization for a patient required <10 min (from time of MIPD platform launch in the “Web Resources” tab in the EHR through calculations to dose selection). Twenty-two (85%) agreed or strongly agreed that the MIPD platform was easy to use. All but one clinical pharmacist agreed or strongly agreed that the ability to access the MIPD platform from within the EHR and the automatic transmission of patient data from the EHR into the MIPD platform were features that helped complete tasks more efficiently. Overall, 21 (81%) agreed or strongly agreed they were satisfied with the CDS tool. The distribution of responses for each question is provided in **Figure 5**.

## Discussion

Through a multidisciplinary team effort, we successfully implemented MIPD in clinical care for vancomycin at our children’s hospital utilizing a commercially available cloud-based CDS tool integrated into the EHR. Adoption of the CDS tool in clinical care was immediate and increased over time, correlating with expansion of the clinical pharmacist led MIPD initiative throughout the hospital. To date, we have used the MIPD CDS tool in >800 neonates and children at our hospital who received vancomycin. MIPD has become the primary approach utilized at our hospital to guide vancomycin dose individualization in patients with 74% of vancomycin treatment courses in which TDM was performed now being supported by the MIPD CDS tool. Accessibility within the clinical workflow and automatic transmission of patient data from the EHR to the CDS tool were key features of the MIPD CDS tool identified by providers. To our knowledge this is the first report demonstrating broad adoption of an EHR integrated MIPD CDS tool into clinical care.

Software platforms that provide MIPD are in essence computerized CDS tools that aid in dose decision-making about a patient. As with any CDS tool, usability of the MIPD platform will be critical to promote adoption (Kannry et al., 2015; Miller et al., 2018), and a simple and easy to use interface that provides the key functionalities and information desired in a reliable and concise manner is needed. However, even a well-designed CDS tool will not be useful if it is not adopted in clinical care. Two of the most important predictors of CDS tool adoption in clinical care are the ability to function within the clinical workflow and the availability at the time of decision making (Bates et al., 2003; Kawamoto et al., 2005; Osheroff et al., 2007; Sittig et al., 2008; Agency for Healthcare Research and Quality, 2009; Miller et al., 2018). The EHR is the central hub of clinical workflow in health-care systems, including where drug dose decisions and ordering are performed (Health IT Dashboard, 2018). Integration of CDS tools within the EHR positions them ideally for ease of access and use by providers within their clinical workflow (Miller et al., 2018). Therefore, EHR integration will be a critical aspect to facilitate adoption of MIPD in clinical care and was a key principle in our local implementation approach. Stand-alone versions of MIPD CDS tools will not be able to provide this level of usability and therefore may inhibit adoption.

EHR integration offers several other advantages. It provides ready access to all of the data about a patient necessary for MIPD and supports the automatic transmission of patient data to the CDS tool. For patients on vancomycin for several days, the number of data elements for a patient can be considerable and manual data entry would be onerous, time consuming, and disruptive to clinical workflow. Minimizing manual data entry is critical as it reduces provider burden, supports timely guidance, and is known to improve CDS tool adoption (Kawamoto et al., 2005; Miller et al., 2018). Our implementation experience supports this notion as access to the MIPD platform from within the EHR and the automatic transmission of patient data were consistently identified by our clinical pharmacists as key features promoting efficiency of use of the CDS tool.

EHR integration of MIPD CDS tools that allow automatic transmission of patient data may also help minimize data entry errors (Séroussi et al., 2012; Gupta et al., 2014). Since patient data are transmitted directly from the EHR, the CDS tool data matches this source. Any data errors that occur result from clinical documentation errors in the EHR (e.g., mis-entered date/time or forgotten documentation of a dose given to patient). CDS dosing tools with a well-designed user interface may actually help identify such errors. For example, when the entire dose and TDM history are presented concisely in chronological order, a mis-documented dose or TDM time can be recognized based on an “unexpected” time interval between events. Functionality that allows such errors to be corrected or flagged in the CDS tool will ensure the appropriate data is used for PK calculations, and any data changes documented and stored for future calculations. Fortunately, EHR technologies such as bar-coded electronic medication-administration systems are helping to reduce medication errors from occurring (Poon et al., 2010).

Clinical pharmacists were an ideal provider group to lead MIPD implementation at our hospital. As experts in the therapeutic use of medications, they have the appropriate skills and training in pharmacokinetics and TDM management to apply the understanding gained to guide dose decision-making within the context of the therapeutic goals for the patient (Carter, 2016). Clinical pharmacists also represent an engaged provider group in terms of valuing dose individualization for patients (Pearce et al., 2018). As a smaller provider pool (~ 60 at LPCH) compared to physicians (> 500 at LPCH), use of the MIPD CDS tool will be more frequent for any given provider allowing proficiency and expertise to develop. In addition, user training and ongoing education are easier to perform among a smaller group of providers. Lastly, the burden of TDM management in the clinical setting often falls on clinical pharmacists, and therefore pharmacokinetic calculations are a pain point in their clinical workflow. CDS tools that facilitate more efficient workflow around pharmacokinetic calculations and interpretation will be valued and EHR integrated CDS dosing tools are desired by clinical pharmacists (Pearce et al., 2018). Several studies have shown pharmacist-led vancomycin dosing and monitoring initiatives result in improved achievement of therapeutic goals in patients (Cardile et al., 2015; Marquis et al., 2015; Momattin et al., 2015).

The MIPD approach has several advantages valuable in clinic care compared to empiric-based PK approaches traditionally used, such as log-linear regression equations. MIPD does not require the assumption of steady-state and utilizes a patient’s entire dosing history in calculations, even if the dose and/or dosing interval recently changed. Traditional empiric PK approaches must often assume the patient is at steady-state at the most recent dose administered. Inappropriate assumptions about steady-state can lead to misinterpretation of a patient’s PK and individual dose need. MIPD can also handle TDM concentrations collected at any time during the treatment course, allowing for more flexibility in the timing of samples. This flexibility is valuable in neonates and young children by allowing TDM samples to be timed with other clinical laboratories, minimizing painful venipunctures (Shah and Ohlsson, 2011) or central line entries that are associated with infections (Callister et al., 2015). The flexibility in timing also allows use of TDM samples drawn at the “incorrect” time, and therefore, no information about the patient is lost. Lastly, other exposure metrics, including the AUC_24_, can be readily calculated with MIPD. This is especially relevant for management of MRSA infections with vancomycin, for which AUC_24_/MIC is the PK-PD metric of choice. The calculations for AUC_24_/MIC can be difficult to perform in the context of clinical workflow, and the trough concentration is often used as a surrogate in clinical care instead. However, the correlation between trough and AUC_24_ are imprecise (Frymoyer et al., 2014; Stockmann et al., 2015; Abulfathi et al., 2018) and dose individualization to optimize the AUC_24_/MIC during clinical care are now recommended. MIPD will allow providers to easily calculate and implement AUC_24_/MIC exposure assessment in therapeutic decision making.

By promoting standardization, MIPD CDS tools can help foster an evidenced-based therapeutic approach across an institution. Such standardization nurtures organizational learning, and as new knowledge is gained, updates to the therapeutic approach can efficiently be implemented in clinical care through the CDS tool. For example, recent reports suggest the unbound fraction of vancomycin is higher in neonates and predicted by serum albumin concentration (Smits et al., 2018). The MIPD approach is agnostic to the underlying PK model and/or exposure target, and updated models that incorporate albumin concentration and fraction unbound into the underlying calculations and exposure considerations can be readily deployed. In this way, MIPD CDS tools can potentially facilitate a learning-health care system with the aim of improving clinical care for each individual patient (Budrionis and Bellika, 2016).

We were able to track population level exposure metrics at our hospital using the data analytics platform that is part of the commercial MIPD software platform used. We used extremely low trough concentrations (< 5 mg/L) or high concentrations (> 20 mg/L) as a countermeasure during implementation, as these levels are almost universally inappropriate regardless of the individual exposure target desired for a patient. By the second TDM, 88% of neonates and 79% of children were within a trough concentration range of 5 to 20 mg/L. If the currently recommended MRSA exposure target of a steady-state AUC_24_/MIC >400 was applied to all of our patients, 78% of neonates and 64% of children achieved this target by the second TDM. Using instead an exposure target of a trough 10 to 20 mg/L, fewer patients would have been considered at target (54% and 44%, respectively). Caution is warranted in interpreting these population level exposure metrics. As a quaternary academic center, our patient population represents a very diverse and sick population. In addition, patient level data are needed to take into account individual considerations (e.g., comorbidities, hospital unit, reasons for vancomycin use, desired exposure target, etc.). Clinical studies evaluating achievement of target exposure, clinical outcomes, and toxicity such as acute kidney injury within defined patient groups will be essential to demonstrate the clinical impact of the MIPD approach, and these investigations are currently underway. The current report serves as an important first step demonstrating feasibility, clinical adoption, and user satisfaction of a vancomycin MIPD approach implemented within an EHR integrated CDS tool.

Our implementation effort represents the experience of a single institution and was designed to take advantage of our local resources and healthcare system. Therefore, the generalizability of our specific approach may be limited. In addition, the individual contribution of each component of our implementation approach in promoting clinical adoption cannot be examined. Nonetheless, our implementation approach incorporated general principles known to promote CDS tool adoption (Bates et al., 2003; Agency for Healthcare Research and Quality, 2009; Kannry et al., 2015; Miller et al., 2018), and these principles can guide implementation strategies at other institutions.

Additional limitations to our implementation approach include the customized integration of the MIPD CDS tool to our hospital’s specific EHR (Epic Systems) build and data architecture. This required the time and effort of clinical informatic specialists skilled at EHR integration, which all centers might not have access to. While the general framework of our approach for integration can be applied at other centers, customization of the API will still be required, and this may limit the ease of replication of our integration approach. Integration approaches taking advantage of developed constructs of data specifications for EHR data (e.g., Health Level Seven International, Fast Healthcare Interoperability Resources) would allow more standardized data exchange between EHR and CDS tool and may result in a more scalable implementation approach (HLA, 2011; Del Fiol et al., 2012; FHIR, 2019).

Implementation of MIPD at our hospital was led by clinical pharmacists, which provided a highly trained and engaged champion of therapeutics to promote adoption. Although the role of clinical pharmacists is expanding across all health care settings (Carter, 2016), not all institutions at this time may have accessibility to clinical pharmacy services. At such institutions, identifying providers who manage vancomycin therapy and evaluate TDM during clinical care will be important to champion change and lead successful implementation and adoption of a MIPD approach.

## Conclusions

Vancomycin MIPD was implemented and adopted into clinical care using a commercially available cloud-based CDS tool integrated into the EHR. EHR integration was fundamental for broad adoption, providing access to the MIPD CDS tool within the clinical workflow of providers and minimizing data-entry burden. Clinical pharmacists led our MIPD implementation and helped promote adoption. While successful implementation of MIPD in clinical care will need to be adapted to each institution’s resources and healthcare system, our framework focused on usability in clinical workflow and local champions to lead may serve as a starting blueprint. Future studies evaluating the impact of MIPD on clinically relevant outcomes are needed.

## Data Availability Statement

The datasets for this article are not publicly available because this was a local quality improvement project. Requests to access the datasets should be directed to AF, frymoyer@stanford.edu.

## Ethics Statement

This project was reviewed by Stanford University’s institutional review board and determined to be local quality improvement work that did not meet the definition of human subjects research and written informed consent was not required as per the local legislation.

## Author Contributions

AF conceptualized and designed the quality improvement project, implemented the quality improvement project, performed analyses and interpretation of data, and drafted the initial manuscript. HS and ShG conceptualized and designed the quality improvement project, implemented the quality improvement project, critically interpreted data, and critically reviewed and revised the manuscript. YZ, LB, JM, BC, and JF helped conceptualize and design the quality improvement project, supported implementation, critically interpreted data, and critically reviewed and revised the manuscript. SrG and RK helped support development of the clinical decision support tool, supported implementation at the hospital, helped with analyses, critically interpreted data, and critically reviewed and revised the manuscript.

## Conflict of Interest

SrG and RK are co-founders and own stock in Insight-RX.

The remaining authors declare that the research was conducted in the absence of any commercial or financial relationships that could be construed as a potential conflict of interest.
